# Evaluating the effects of various ethanolic medicinal plant extracts on metastatic breast cancer proliferation, invasion, and expression of a novel potential drug target; CD82 metastatic suppressor protein, and on in vivo angiogenesis using the *ex ovo* yolk sac membrane (YSM) assay

**DOI:** 10.1007/s00432-024-05751-0

**Published:** 2024-05-16

**Authors:** Samantha Loggenberg, Danielle Twilley, Namrita Lall

**Affiliations:** 1 Department of Plant and Soil Sciences, University of Pretoria, Pretoria, 0002 South Africa; 2 School of Natural Resources, University of Missouri, Columbia, MO 65211 USA; 3 College of Pharmacy, JSS Academy of Higher Education and Research, Mysuru, Karnataka 570015 India; 4 Bio-Tech Research and Development Institute, University of the West Indies, Kingston, Jamaica

**Keywords:** Metastatic breast cancer, CD82 metastatic suppressor protein, *Buddleja saligna* Willd., *Combretum apiculatum* Sond., *Persicaria senegalensis* (Meisn.) Soják, Tumour angiogenesis

## Abstract

**Purpose:**

Breast cancer metastasis relies on cellular invasion and angiogenesis facilitated by the downregulation of metastatic suppressor proteins like Cluster of Differentiation 82 (CD82). Currently, no medicines target multiple systems to prevent metastatic progression through CD82 upregulation. This study screened for plant extracts displaying effects on cell proliferation, invasion, and CD82 expression in breast cancer cells, and in vivo angiogenesis, and further correlated between the biological activities and effect on CD82 expression.

**Methods:**

Seventeen ethanolic plant extracts were screened for their effect on cell proliferation (against MDA-MB-231 and MCF-7 breast cancer and Hek293 kidney cells), cell invasion and effect on CD82 expression in metastatic MDA-MB-231 cells. Selected extracts were further evaluated for in vivo anti-angiogenesis.

**Results:**

Extracts displayed varying antiproliferative activity against the different cell lines, and those that showed selectivity indexes (SI) > 0.5 against MDA-MB-231 were selected for anti-invasion evaluation. *Buddleja saligna* Willd. (BS), *Combretum apiculatum* Sond. (CA), *Foeniculum* vulgare, *Greyia radlkoferi*, *Gunnera perpensa* and *Persicaria senegalensis* (Meisn.) Soják (PS) displayed 50% inhibitory concentration (IC_50_) values of 44.46 ± 3.46, 74.00 ± 4.48, 180.43 ± 4.51, 96.97 ± 2.29, 55.29 ± 9.88 and 243.60 ± 2.69 µg/mL, respectively against MDA-MB-231, and compared to Hek293 showed SI of 0.9, 0.7, 1.4, 1.1, 2.2 and 0.5. Significant invasion inhibition was observed at both 20 and 40 µg/mL for BS (94.10 ± 0.74 and 96.73 ± 0.95%) and CA (87.42 ± 6.54 and 98.24 ± 0.63%), whereas GR (14.91 ± 1.62 and 41 ± 1.78%) and PS (36.58 ± 0.54 and 51.51 ± 0.83%), only showed significant inhibition at 40 µg/mL, and FV (< 5% inhibition) and GP (10 ± 1.03 and 22 ± 1.31%) did not show significant inhibition at both concentrations. Due to the significant anti-invasive activity of BS, CA and PS at 40 µg/mL, these extracts were further evaluated for their potential to stimulate CD82. BS showed significant (*p* < 0.05) reduction in CD82 at 20 and 40 µg/mL (13.2 ± 2.2% and 20.3 ± 1.5% decrease, respectively), whereas both CA and PS at 20 µg/mL increased (*p* < 0.05) CD82 expression (16.4 ± 0.8% and 5.4 ± 0.6% increase, respectively), and at 40 µg/mL significantly reduced CD82 expression (23.4 ± 3.1% and 11.2 ± 2.9% decrease, respectively). Using the yolk sac membrane assay, BS (59.52 ± 4.12 and 56.72 ± 3.13% newly formed vessels) and CA (83.33 ± 3.17 and 74.00 ± 2.12%) at both 20 and 40 µg/egg showed significant (*p* < 0.001) angiogenesis inhibition, with BS showing statistical similar activity to the positive control, combretastatin A4 (10 nmol/egg), whereas PS only displayed significant (*p* < 0.001) angiogenesis stimulation at 40 µg/egg (120.81 ± 3.34% newly formed vessels).

**Conclusion:**

BS exhibits antiproliferative, anti-invasive, and anti-angiogenic activity despite inhibiting CD82, suggesting an alternative mode of action. CA at 20 µg/mL shows moderate anti-invasive and anti-angiogenic potential by stimulating CD82, while at 40 µg/mL it still displays these properties but inhibits CD82, suggesting an additional mode of action. PS, with the least antiproliferative activity, stimulates CD82 and inhibits angiogenesis at 20 µg/mL but inhibits CD82 and increases angiogenesis at 40 µg/mL, indicating CD82 targeting as a major mode of action. Future studies should explore breast cancer xenograft models to assess the extracts’ impact on CD82 expression and angiogenesis in the tumor microenvironment, along with isolating bioactive compounds from the extracts.

## Introduction

In 2018, the Cancer Association of South Africa (CANSA 2019) stated that breast cancer is the most prevalent cancer-type diagnosed in South African women, with an incidence rate of 13.1% and a lethality rate of 8.2%. Furthermore, cancers which undergo metastasis are responsible for over 90% of cancer fatalities and approximately 20–30% of diagnosed cases of breast cancer progress to metastatic malignancy. Cancers metastasise when the cancerous cells disseminate from the site of origin and spread to other tissues of the body, leading to the development of distal tumour sites (Seyfried and Huysentruyt 2014). Metastatic breast cancers are the most difficult cancers to treat exhibiting a high reoccurrence rate of 7–11% over a 5 year period (American Society of Clinical Oncology (ASCO) 2022). Most modern anticancer treatments, such as chemotherapies, are effective in killing cancerous cells but are indiscriminate of normal somatic cells (O’Shaughnessy 2005). Patients undergoing chemotherapies may experience prolonged negative side-effects, such as weight and hair loss (Gray et al. 2021). Therefore, identification of potential treatments options should focus on pathways which may aid in supressing metastatic developments in breast cancers. Factors contributing to metastatic progression of the disease include the invasive development of cancer cells, the degradation of the extracellular matrix (ECM) basement membrane between tissues and tumour angiogenesis (He et al. 2015).

Current antimetastatic therapeutics are limited as most treatments aim to kill the cancer cells for direct shrinkage of both initial and distal tumours, or to eliminate free-floating micro metastases (cancer cells, or cancer masses which have successfully disseminated from the cancer origin site) (Weber 2021). Furthermore, current anticancer treatments may be ineffective in total eradication of the malignancy within the body, whilst additionally killing healthy somatic cells (Rosel et al. 2017). As most common drugs display singular or broad-spectrum target pathways, there are currently no drugs available which target multiple cellular systems which suppress factors associated with the initial development of metastatic breast cancer (Qin et al. 2014). Therefore, research into novel treatments should consider investigating molecular targets which affect multiple factors contributing to metastatic potential, such as angiogenesis, cell proliferation and cell invasion, facilitated by the upregulation of metastatic suppressor proteins naturally present within cells (Bossung and Harbeck 2010). A potential novel drug candidate, the transmembrane Cluster of Differentiation 82 (CD82) metastatic suppressor protein, which plays a crucial role in regulating multiple cellular pathways involved in angiogenesis, cell invasion and cell proliferation, therefore, provides a potential novel drug target (Al-Khater et al. 2021; Liu et al. 2012; Wei et al. 2014; Zhu et al. 2017). The CD82 metastatic suppressor protein is expressed in all somatic cells, however, various studies show that the protein is under-expressed in both invasive and non-invasive breast cancer (Al-Khater et al. 2021; Singh et al. 2016; Wang et al. 2019a, b). To date, there are currently no drugs which target the upregulation of the CD82 protein in breast cancer to prevent the initial stages facilitating metastatic progression of the disease.

The most commonly known anticancer drugs available, such as paclitaxel and vinca-alkaloids, were derived from medicinal plants. However, there is limited research which closes the gap between the use of medicinal plants to treat cancers and their effect on the progression of metastatic developments of breast cancer. The following medicinal plant candidates were selected for this study based on previously displayed in vitro antiproliferative activity (Table 1).

**Table 1:** Medicinal plants and their in vitro antiproliferative activity

Plant species	Plant family	In vitro antiproliferative activity/potential	Indigenous region	Reference
*Acokanthera oppositifolia* (Lam.) Codd	Apocynaceae	A methanolic leaf extract exhibited IC_50_^a^ values of 2.5 ± 0.06 and 2.85 ± 0.08 μg/mL against CCRF-CEM and CEM/ADR5000 leukaemia cells, respectively	Southern Africa; Kenya, Southern Democratic Republic of the Congo, and South Africa	Shirinda et al. (2019)
*Afrocarpus falcatus* (Thunb.) C.N.Page	Podocarpaceae	Nagilactones isolated from the roots: 16-hydroxynagilactone F and 2β,16-dihydroxynagilactone F, displayed IC_50_ values of 0.6 ± 0.4 and 1.1 ± 0.5 μM against HT-29 human colorectal adenocarcinoma cells	South-eastern Africa; Malawi, Mozambique, South Africa, and Eswatini	Addo et al. (2015)
*Buddleja saligna* Willd.	Scrophulariaceae	Ethanolic extracts of the leaves and stems exhibited an IC_50_ of 31.80 ± 0.35 µg/mL against UCT-MEL-1 human malignant melanoma cells	Southern Africa; South Africa and Zimbabwe	Twilley et al. (2021)
*Bulbine frutescens* (L.) Willd.	Asphodelaceae	Methanolic and hexane leaf extracts displayed IC_50_ values of 4.88 and 13.12 μg/mL against MDA-MB-231, respectively, and IC_50_ values of 6.35 and 12.08 μg/mL against T47D breast cancer cells, respectively	Southern Africa; South Africa, Lesotho, Eswatini	Prakash et al. (2019)
*Combretum apiculatum* Sond.	Combretaceae	An ethanolic leaf extract exhibited an IC_50_ of 56.40 ± 6.11 and 90.53 ± 4.94 µg/mL against A431 squamous cell carcinoma and UCT-MEL-1 cells, respectively	Tropical Eastern and Southern Africa; Kenya, Tanzania, Angola, Botswana, Malawi, Mozambique, Namibia, Zambia, Zimbabwe and South Africa	Maphutha et al. (2021)
*Euphorbia cooperi* N.E.Br. ex A. Berger	Euphorbiaceae	A chloroform fraction from a hexane extract displayed antiproliferative activity against MCF-7 breast cancer cells, HepG2 liver carcinoma cells, HeLa cervix cancer cells and HFB4 melanocytes, with IC_50_ values of 4.23 ± 0.08, 10.8 ± 0.74, 26.6 ± 2.1 and 15.6 ± 1.15 μg/mL, respectively	South Africa	Islam and Ahmed (2015)
*Euphorbia ingens* E.Mey. ex Boiss.	Euphorbiaceae	Euphol, isolated from the latex, displayed IC_50_ values of 0.26, 0.22 and 0.13 mM against T47D ductal carcinoma	Southern Africa; South Africa, Mozambique and Zimbabwe	Calvin et al. (1979)
*Foeniculum vulgare* Mill.	Apiaceae	A methanolic seed extract exhibited an IC_50_ of 50 ± 0.03 μg/mL against MCF-7 cells	Southern Europe; Mediterranean coast regions	Mohamad et al. (2011)
*Galenia africana* L.	Aizoaceae	An ethanolic leaf extract exhibited IC_50_ values of 114.0, 130.5 and 159.81 μg/mL against MCF-7, MDA-MB-231 cells and MCF-12A normal breast cells, respectively	South Africa	Mohamed et al. (2020)
*Gunnera perpensa* L.	Gunneraceae	Z-venusol isolated from the leaves exhibited an IC_50_ of 53.7 μg/mL against MCF-7 cells	Southern-Africa: Sudan, Ethiopia, Zaire, Rwanda, Uganda, Kenya, Tanzania, Zimbabwe, Mozambique and South Africa	Mathibe et al. (2016)
*Greyia radlkoferi* Szyszył.	Francoaceae	An ethanolic leaf extract inhibited cell proliferation and migration in HaCat keratinocytes using the scratch assay (showing percentage closure of 60.15 ± 1.41%, and 49.52 ± 1.43% at concentrations of 50 and 100 μg/mL, respectively	South Africa	Loggenberg et al. (2022)
*Myrothamnus flabellifolius* Welw.	Myrothamnaceae	Methanolic and petroleum ether extracts exhibited IC_50_ values of 62.5 ± 0.40 and 250 ± 0.40 μg/mL, against HL-60 leukemia cells, respectively	South Africa	Dhillon et al. (2014)
*Myrsine africana* L.	Myrsinaceae	Embelin, isolated from the leaves and fruit, displayed IC_50_ values of 6.04 and 4.45 μM against MCF-7 and MDA-MB-231, respectively	South Africa	Lall et al. (2017)
*Persicaria senegalensis* (Meisn.) Soják	Polygonaceae	Methanolic leaf and seed extracts displayed antiproliferative activity against PC3 prostate cancer (IC_50_ values of 2.3 ± 0.03 and 3.5 ± 0.06 μg/mL, respectively) and Caco-2 colon cancer cell lines (2.0 ± 0.03 and 1.5 ± 0.03 μg/mL, respectively)	Africa and Arabian Peninsula	Mohamed et al. (2018)
*Plectranthus ecklonii* Benth.	Lamiaceae	Parvifloron D, isolated from the whole plant, displayed IC_50_ values of 2.48 and 34.3 ± 4.1 µM against MDA-MB-231 and MCF-7 breast cancer cells, respectively	South Africa	Burmistrova et al. (2015)
*Tylosema esculentum* (Burchell) A. Schreiber	Fabaceae	Potent antiproliferative compounds, such as catechin, rutin, hesperidin, naringin, myricetin, naringenin, kaempferol and gallic acid, have been isolated from the husk and seeds material	Southern Africa; Namibia, Botswana, and South Africa	Chingwaru et al. (2011)

^a^50% inhibitory concentration

Despite the literature presented in Table 1, there is a lack of previous research which evaluates these plants for their potential in vitro activity against factors suggested to facilitate cancer metastasis, such as cell invasion, reduced expression of CD82, and angiogenesis. Furthermore, there are no studies which identify the correlation between the anti-invasive effects of plant extracts and the expression of CD82 protein in breast cancer cells.

This study aimed to evaluate the antiproliferative and anti-metastatic potential of seventeen (17) ethanolic (EtOH) extracts of the 16 medicinal plants, mentioned in Table 1, against breast cancer cells. Thereafter, the mechanism of action was evaluated by determining their potential to upregulate CD8, which was correlated to the potential to inhibit in vivo angiogenesis. Furthermore, this study aimed to identify whether the extracts effect on CD82 expression would correlate with the antiproliferative, anti-invasion and anti-angiogenic activity, in order to identify additional plant candidates which may show antimetastatic potential against breast cancer through stimulation of CD82, and therefore, may be evaluated further in pre-clinical studies.

## Materials and methods

### Materials and reagents

The MDA-MB-231 and MCF-7 breast cancer cell lines, and human embryo kidney cell line Hek293, were purchased from Separations Scientific SA (Pty) Ltd. (Roodepoort, South Africa). Cell culture reagents, including Dulbecco’s Modified Eagle Medium (DMEM), DMEM/Nutrient Mixture F-12 Ham (1:1) (DMEM/F-12), trypsin–EDTA (0.25%), fetal bovine serum (FBS), phosphate buffer saline (PBS) and antimicrobials, such as penicillin, streptomycin and amphotericin B, and PrestoBlue® viability reagent were supplied by ThermoFisher Scientific (Johannesburg, South Africa). Sterile cell culture flasks and multi-well plates were purchased from Lasec South Africa (Pty) Ltd (Midrand, South Africa). Paclitaxel (purity > 95%), Doxorubicin (purity > 98%), sodium azide (> 95%) and all other reagents were supplied by Sigma Chemicals Co. (St. Louis, MO, USA). The Chemicon® QCM ECMatrix Cell Invasion Assay, 24-well (8 µm), Fluorimetric Kit (Cat # ECM554) was bought from Merck (Pty) Ltd (Pretoria, South Africa). The PE anti-human CD82 antibody (Cat # 342104) was obtained from Biocom Africa (Pty) Ltd (Centurion, South Africa). Three-day old fertilized White Leghorn Chicken, of specified pathogen free (SPF) status, eggs were purchased from Avi-farms (Pty) Ltd. (Centurion, South Africa).

### Plant material collection and extraction

Ethanolic extracts were prepared from the dried plant material, with slight variation, for each of the plants listed in Table 2. For all plants, except *F. vulgare* and *M. flabellifolius*, fresh plant material was collected during summer months (beginning of December to end of February) from the Manie van der Schijff Botanical Garden (25.75194°S 28.22889°E), or Experimental Farm (25.7494°S, 28.2544°E), at the University of Pretoria. Herbarium specimen numbers were prepared and submitted at the H.G.W.J. Schwikert Herbarium (University of Pretoria, South Africa) (Table 2). Collected fresh plant material were stored in a dark room, free from damp, and allowed to air-dry until the plant material was completely dried. Air-dried and homogenised plant material of *F. vulgare* and *M. flabellifolius* was purchased from MuthiFuthi™ (KwaZulu-Natal, South Africa) in June of 2014, and stored at room temperature until extract preparation, February of 2021. Briefly, the collected plant material was extracted in absolute ethanol (± 1:4 ratio between plant material (kg): EtOH (L)) for approximately 48 h per extraction. For *E. cooperi* and *E. ingens*, fresh fleshy whole stems were first homogenized by blending before being extracted in absolute ethanol. Thereafter, the plant-ethanol mixtures were filtered with a Buchner funnel (using No 3 Whatman filter paper) and the filtrate was then concentrated using a Rotary evaporator (Bϋchi, Switzerland). Plant material was re-extracted and the ethanol solvent was collected until clear. If necessary, liquid-like extracts were further freeze-dried using a freeze dryer (ICHR101530, Alpha 1-2 Ldplus (Lasec SA (Pty) Ltd.), to obtain a powdered extract. All obtained extracts were stored at −4 °C until use for subsequent bioassays.

**Table 2:** Accession numbers of the medicinal plant candidates and the percentage extraction yield obtained for each extract

Plant species	Accession number	Plant part used	Weight (kg)	Amount of ethanol used for extraction (L)	Percentage yield (%)
*Acokanthera oppositifolia* (Lam.) Codd	129342	Dried leaves	1.28	2.3	23.50
*Afrocarpus falcatus* (Thunb.) C.N.Page	96409	Dried leaves	2.00	1.5	12.30
*Buddleja saligna* Willd.	122167	Dried leaves and stems	1.66	9.0	14.50
*Bulbine frutescens* (L.) Willd.	122179	Whole fresh leaves and gel	0.46	0.3	11.85
*Combretum apiculatum* Sond.	124380	Dried leaves	0.43	1.7	7.40
*Euphorbia cooperi* N.E.Br. ex A. Berger	128546	Fresh fleshy stems and latex	1.22	1.4	4.30
*Euphorbia ingens* E.Mey. ex Boiss.	128545	Fresh fleshy stems and latex	1.34	5.4	6.20
*Foeniculum vulgare* Mill.	Plant material received from MuthiFuthi™	Dried aerial parts	0.45	1.8	11.30
*Galenia africana* L.	93723	Dried leaves	2.50	10.0	4.50
*Gunnera perpensa* L.	120010	Dried leaves	1.58	6.0	14.6
*Greyia radlkoferi* Szyszył.	122434	Dried leaves	0.20	2.0	6.22
*Myrothamnus flabellifolius* Welw.	Plant material received from MuthiFuthi™	Dried aerial parts	0.50	2.0	15.6
*Myrsine africana* L.	MA-S-2013-1	Dried leaves	2.30	5.0	5.34
*Persicaria senegalensis* (Meisn.) Soják	122180	Leaves	0.25	1.0	6.57
*Plectranthus ecklonii* Benth.	122337	Leaves	0.36	1.4	8.91
*Tylosema esculentum* (Burchell) A. Schreiber	130879	Leaves and husk, separately	5.00	5.0	6.25

### Cell culture

#### Culture growth and maintenance

The MCF-7 (passage # 20) and Hek293 (passage # 20) cell lines were maintained in DMEM, whereas the MDA-MB-231 (passage # 25) cell line was maintained in DMEM/F-12, where all media was supplemented with 10% heat-inactivated foetal bovine serum, 1% antibiotics (penicillin at 100 U/mL and streptomycin at 100 μg/mL), and 1% amphotericin B (250 μg/mL), at 37°C and 5% CO_2_. After reaching an 80% confluency, the cells were sub-cultured after 5 min treatment with trypsin–EDTA (0.25% trypsin containing 0.01% EDTA).

#### Antiproliferative assay

The antiproliferative assay was performed according to the method described by Lall et al (2013). Briefly, in sterile 96-well micro-titre plates, cells were seeded at a concentration of 1 × 10^5^ cells/mL (10,000 cells/well) with 100 µL of cell suspension per well, and the plates were incubated for 24 h to allow cell attachment to occur. A media without cells control served as the 0% cell viability, whereas a media control with cells acted as the 100% cell viability control. Dimethyl sulfoxide (DMSO) was included as a as a vehicle control (1%) and as a toxic inducer being tested at final concentrations ranging from 0.625–20%. Stock concentrations of the extracts were prepared in DMSO at a concentration of 40 mg/mL. The extracts were serially diluted in complete media and tested at final concentrations ranging from 3.13–400 μg/mL. Doxorubicin served as the positive drug control, with a stock concentration being prepared in DMSO at 2 mg/mL, and was further tested at final concentrations of 0.032–1 µg/mL. A final testing volume of 200 µL per well was used for all wells. The plates were incubated for a further 72 h at 37 °C and 5% CO_2_. Thereafter, 20 µL of PrestoBlue® viability reagent was added to all wells, followed by an additional 2 h incubation to allow for a colour change to occur. The fluorescence of the resulting colour complex was read at an excitation wavelength of 560 nm and an emission wavelength of 590 nm, using a Victor® Nivo™ Multimode Microplate Reader (PerkinElmer South Africa (Pty) Ltd.). The percentage cell viability was determined using the following formula:

$$\%\ {\text{Cell viability}} = \left[ {\frac{{\left( {{\text{A}}_{{{\text{treatment}}}} { } - {\text{ A}}_{{{\text{blank}}}} } \right)}}{{\left( {{\text{A}}_{{{\text{control}}}} { } - {\text{ A}}_{{{\text{blank}}}} } \right)}}} \right] \times 100 \%$$

where A = Fluorescence, A_blank_ = fluorescence of the 0% control (media without cells), A_control_ = fluorescence of vehicle control (1% DMSO), and A_treatment_ = fluorescence of sample. The 50% inhibitory concentration (IC_50_) of all plant extracts, doxorubicin and the DMSO toxic inducer control was determined using the GraphPad Prism Version 4.0 software (San Diego, California, USA).

The IC_50_ values obtained against the cancerous MDA-MB-231 and MCF-7 cell lines were compared to the IC_50_ values obtained against the non-cancerous Hek293 cell line in order to determine the selectivity index (SI) values for each sample. The following formula was used to calculate the SI values for each sample:

$$SI\;value = \frac{{IC_{50}\;non{ - }cancerous\;cells}}{{IC_{50}\;cancer\;cells}}$$

where SI = Selectivity index, IC_50_ non-cancerous = IC_50_ value of the sample against the non-cancerous Hek293 cell line, and IC_50_ cancerous = IC_50_ value of the sample against the cancerous MDA-MB-231 or MCF-7 cell line.

#### Cell invasion assay

The cell invasion assay was performed using the invasive MDA-MB-231 cell line using the Chemicon® QCM ECMatrix Cell Invasion Assay, 24-well (8 µm), Fluorimetric Kit according to the manufacturers protocol. Briefly, the kit included sterile 24-well plates with specialised well inserts to separate the upper and lower well chambers with a Matrigel™ layer to act as an ECM layer through which the cells may migrate. Twenty-four hours prior to conducting the assay, MDA-MB-231 cells (at 80% confluency) were serum-starved by incubation in incomplete media (DMEM/F-12 supplemented with 1% antibiotics and 1% amphotericin B, without FBS). Thereafter, the cells were detached with trypsin–EDTA and resuspended in incomplete media to reach a concentration of 1 × 10^6^ cells/mL. Prior to this, 300 µL of incomplete media at room temperature was added to the 24-well inserts of the kit for the Matrigel™ layer to rehydrate for 30 min at room temperature. Thereafter, the remaining media was removed from the 24-well inserts, without disturbing the Matrigel layer, and was replaced with 300 µL of cell suspension (3 × 10^5^ cells per well insert) and sample was simultaneously added to the upper chamber. The inserts were subsequently placed into the 24-well plates provided with the kit, and 500 µL of complete media (DMEM/F-12 supplemented with 10% FBS, to act as the chemoattractant to stimulate invasive potential of the cells) was added to each bottom well chamber. Stock concentrations of selected extracts were prepared in serum-free media (4 mg/mL, 20% DMSO) and tested at final concentrations lower than their IC_50_ values; 20 µg/mL (0.1% DMSO) and 40 µg/mL (0.2% DMSO). Media controls with and without cells served as the 100% cell invasion and 0% cell invasion controls, respectively and an additional media control with cells and no FBS in the bottom chamber served as the chemoattractant control. A stock concentration of taxol, the positive drug control, was prepared (40 µg/mL in 0.02% DMSO) and tested at two final concentrations of 0.25 (0.001% DMSO) and 0.5 µg/mL (0.002% DMSO). A DMSO vehicle control was included in the assay and tested at a final concentration of 0.2%. Invasive breast cancer MDA-MB-231 cells were treated with the ethanolic extracts of BS, CA, FV, GR, GP, and PS, at two concentrations (20 and 40 µg/mL). The plates were incubated for 72 h and the cells, which had successfully invaded the Matrigel filter, were quantified in relation to the vehicle (0.2% DMSO) control to obtain percentage cell invasion (%) values, which were used to calculate percentage inhibition of cell invasion by each sample. After incubation, the cell suspension was aspirated from the upper chamber inserts, which were subsequently removed and placed into clean 24-wells filled with 225 µL of cell detachment solution (at room temperature) provided with the assay kit, and the plates were further incubated for 30 min at 37 °C, with slight tilting of the plates every 5 min to facilitate cell detachment from the underside of the upper inserts. Lysis buffer/dye solution was prepared according to the manufacturer’s protocol (1:75, CyQuant GR Dye in lysis buffer). The inserts were removed, and 75 µL of lysis buffer/dye was added to the wells containing cell detachment solution, and the plates were subsequently incubated for 15 min at room temperature to allow attachment of the dye to cellular DNA. In a sterile 96-well microtitre plate, 100 µL of the incubated mixture was added in duplicate to separate wells and diluted in 100 µL of PBS (1:1). The fluorescens of the wells was then measured at an excitation wavelength of 480 nm and an emission wavelength of 520 nm, using a Victor® Nivo™ Multimode Microplate Reader (PerkinElmer South Africa (Pty) Ltd.). The percentage inhibition of cell invasion was determined using the following formula:

$$\% \ {\text{Inhibition of cell invasion}} = 100 \% - \left[ {100 \% \times \left[ {\frac{{\left( {{\text{A}}_{{{\text{treatment}}}} - {\text{A}}_{{{\text{blank}}}} } \right)}}{{\left( {{\text{A}}_{{{\text{control}}}} - {\text{A}}_{{{\text{blank}}}} } \right)}}} \right]} \right]$$

where A = Fluorescence, A_blank_ = fluorescence of the 0% control (media without cells), A_control_ = fluorescence of vehicle control (0.2% DMSO), and A_treatment_ = fluorescence of sample.

### Treatment and preparation of cells for flow cytometry, and quantification of CD82 metastasis protein

In separate sterile T75 cell culture flasks, MDA-MB-231 cells were seeded at 5 × 10^4^ cells/mL (1 × 10^6^ cells per flask) in complete media and incubated for 24 h to allow cell attachment to occur. Thereafter, the media was removed, and the cells were washed with PBS before 30 mL of fresh complete media was added, either supplemented with or without sample. Stock concentrations of selected extracts were prepared in DMSO at concentrations of 20 and 40 mg/mL (in 10% DMSO), which was added to the culture flasks (300 µL) to final concentrations of 20 µg/mL (0.05% DMSO) or 40 µg/mL (0.1% DMSO). A flask with media and cells and a flask with 0.1% DMSO were included as a media and a vehicle control, respectively. The cell culture flasks were further incubated for 72 h before preparation for flow cytometry. After incubation, the media was removed, and the cells were washed and detached from the flasks. A cell count was done for each flask using a Countess 3 Automated Cell Counter (ThermoFisher Scientific (Johannesburg, South Africa)) to ensure viability above 90%. To prepare the cell suspensions for flow cytometry, Cell Staining Buffer (CSB) was prepared (1% sodium azide and 10% FBS in PBS) and cells were suspended in 15 mL CSB at a concentration of 1 × 10^6^ cells/mL in 50 mL falcon tubes. The cell suspensions were centrifuged at 980 rpm for 5 min, the supernatant was discarded, and the pellet was resuspended in 1 mL of CSB to wash the cells. Thereafter, 100 µL aliquots of each sample (in triplicate) were pipetted into separate 2 mL Eppendorf tubes (5 × 10^5^ cells per tube). Five microlitres of PE anti-human CD82 antibody was added to each Eppendorf tube and further incubated in the dark for 30 min at room temperature. A separate aliquot without addition of antibody was included as the unstained control. Thereafter, the cell suspensions were centrifuged, the supernatant was removed, and the pellet was resuspended in 100 µL CSB. The previous step was repeated to wash the cells and the cell pellets were resuspended in 500 µL CSB (5 × 10^5^ cells per tube) and immediately used for flow cytometry.

The effect of BS, CA and PS, on the expression of CD82 in MDA-MB-231 cells was determined using flow cytometry. Samples were acquired using a BD Accuri™ C6 Plus Flow Cytometer, BD Biosciences (San Diego, CA, United States) preconfigured with a green (533/30) standard optical filter and using the FL2-A sensors. The number of events recorded was set to 5000 and the flow rate was set to slow. The data were analysed, appropriate gating of cell populations was set and cell count versus fluorescens intensity histograms were produced using the FlowJo software (Version 10.8.1.8).

For all samples and the vehicle controls, the percentage change of CD82 population fluorescence was determined as the percentage difference between the peak FL2 fluorescence intensity (measured in arbitrary units (AU)) of untreated (stained) cells and treated cells (stained). As the FL2 fluorescence was detected as a logarithmic scale, the difference between the modes in each treatment was calculated as follows:

$$Percentage\;change\;in\;CD82 \left( \% \right) = 100\left( {\frac{{\left( {\log (FL2_{treated} )} \right) - \left( {\log (FL2_{untreated} )} \right)}}{{\left( {\log (FL2_{untreated} )} \right)}}} \right)$$

where FL2 = Peak fluorescence intensity (AU) of stained cell populations; FL2_treated cells_ = Fluorescence intensity (AU) of cells treated with sample; and FL2_untreated cells_ = Fluorescence intensity (AU) of untreated cells.

### *Ex ovo* egg yolk sac membrane (YSM) assay

The YSM was conducted to evaluate the effect of BS, CA and PS on in vivo angiogenesis and was performed according to the methods described by Dohle et al. (2010) and Twilley et al (2022), with modifications. Three-day old fertilized White Leghorn chicken eggs were incubated for 96 h at 37 °C and 90% (v/v) relative humidity. To prevent adherence of the yolk-sac to the shell during incubation, the eggs were gently turned three times daily. After incubation, the eggs were opened into weigh boats (L89 × W89 × H25 mm) with the yolk-sac and blood vessels facing upwards. The opened eggs were then covered with weigh boats of equal dimensions with hole punctures on the top and the eggs were further incubated for an additional 24 h to allow for stabilization of the YSM. Thereafter, autoclaved filter paper (Whatman No. 1) disc punch-outs (6 mm in diameter) were placed on the blood vessels and 16 µL of sample was pipetted directly onto the filter paper. Each egg was treated with three to five samples (depending on the size of the yolk sac), which included at least one control (PBS or DMSO vehicle control. The extracts of BS, CA and PS were tested at 20 and 40 µg/egg, whereas the positive control combretastatin A4 was tested at 10 nmol/egg. The controls included PBS and two vehicle controls, 0.78 and 1.56% DMSO, which acted as the vehicle controls for 20 and 40 µg/egg, respectively. After sample was added to the filter paper discs, the eggs were covered and further incubated for a further 24 h at 37 °C and 90% (v/v) relative humidity. Images of the blood vessels surrounding the filter paper discs, loaded with the samples and controls, were taken at 0 and 24 h of incubation using a digital USB microscope camera (Opti-Tekscope OT–V1). In chick embryos, the development of pain receptors begins after day seven of growing, therefore, each YSM assay was terminated within 7 days (from first incubation). After the 24 h images were taken, the embryos (inside the closed weigh boats) were frozen for 30 min at −80 °C and thereafter were immediately autoclaved and disposed of as biohazard waste. Images were analyzed using the ImageJ Version 1.53e Software. Briefly, the colour channels of the images were split and the green channel image was used for further processing. Noise reduction was applied (at a radius of 5.0 pixels) to remove dark outliers (threshold set to 50) and the contrast was enhanced by 1.0. The ‘find maxima’ tool was applied with a prominence of 6.0, excluding edge maxima and the output type was set to ‘segmented articles’ to obtain a skeletal image of the YSM. The number of branches were measured using the Analyze Skeleton plugin and the point selection counting tool was used to count the number of tertiary arterioles branching from the main veins. The number of nascent blood vessels was calculated by normalizing the number of nascent tertiary arterioles in the sample treated YSM to those of their respective vehicle controls (0.78 and 1.56% DMSO).

### Statistical analysis

The antiproliferative assays were performed in triplicate with three independent experiments (n = 3). The cell invasion assay was performed in duplicate with two separate experiments (n = 2), and three reads per sample, whereas quantification of CD82 expression was performed in triplicate with three separate experiments (n = 3). The YSM assay was performed in duplicate with two independent experiments (n = 2). The results were displayed as mean ± standard deviation. The IC_50_ from the antiproliferative assay were calculated using non-linear regression analysis of the sigmoidal dose–response curves with constraints set at 100 (top) and 0 (bottom) using GraphPad Prism Version 4.0 software. One-way ANOVA using comparison to the positive drug (+) controls or respective DMSO vehicle controls, followed by Dunnett’s or Tukey’s multiple comparison test (as indicated in the results section) using GraphPad Prism Version 4.0 software, with *p < 0.05 and **p < 0.01 indicating statistical significance, unless otherwise stated.

## Results

### Antiproliferative activity and selectivity

**Fig. 1:**
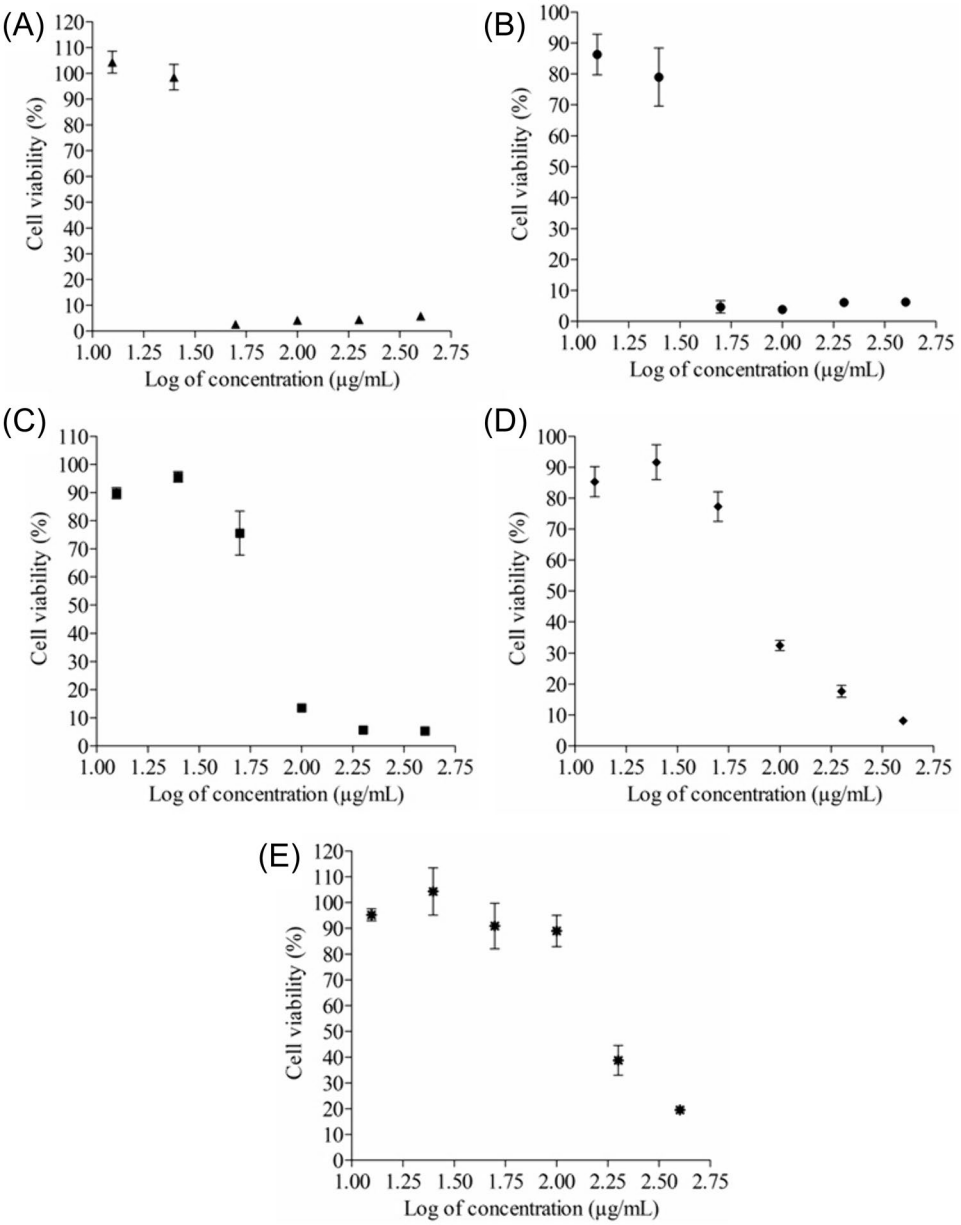
Dose–response curves of MDA-MB-231 breast cancer cells treated with ethanolic extracts of **A** *Buddleja saligna*, **B** *Combretum apiculatum*, **C** *Foeniculum vulgare*, **D** *Greyia radlkoferi*, **E** *Gunnera perpensa*, and **F** *Persicaria senegalensis* after 72 h of treatment

The ethanolic extracts were evaluated for their antiproliferative activity against MDA-MB-231 invasive and MCF-7 non-invasive breast cancer cell lines, and the non-cancerous Hek293 human embryonic kidney cell line, to determine the 50% inhibitory concentration (IC_50_) values after 72 h of treatment (Table 3, Fig. 1). The SI values were calculated for the extracts which displayed IC_50_ values against the cancerous and non-cancerous cell lines. Extracts which displayed SI values, comparing IC_50_ values between the invasive MDA-MB-231 breast cancer cell line and non-cancerous Hek293 human kidney cell line, larger than 0.5 were selected for further testing (SI ≥ 0.5), as this was the lowest SI value displayed by the positive control. Extracts which displayed SI values lower than 0.5 were excluded for further testing. Therefore, the ethanolic extracts of *B. saligna*, *C. apiculatum*, *F. vulgare*, *G. radlkoferi*, *G. perpensa*, and *P. senegalensis* were selected for their anti-invasive activity. To ensure that the viability of the cells was not compromised by the addition of 1% DMSO against each cell line, the cell viability of the 1% DMSO vehicle control was calculated in relation to the viability of the respective media only control (set as 100% viability), and subsequently displayed minimal variation in cell viability against MDA-MB-231, MCF-7 and Hek293 cells (displaying cell viability values of 100.03 ± 3.94, 98.57 ± 4.00, and 99.05 ± 2.11%, respectively).

**Table 3:** The 50% inhibitory concentration (IC_50_) values (µg/mL) and selectivity index (SI) values of the ethanolic extracts against breast cancer cells (MDA-MB-231 and MCF-7) and human embryonic kidney (Hek293) cells, after 72 h of treatment

Ethanolic extract	MDA-MB-231	MCF-7	Hek293	MDA-MB-231	MCF-7
	IC_50_^b^ ± SD^c^ (µg/mL)			SI^d^ value	
*Acokanthera oppositifolia*	> 400	229.87 ± 7.70	< 12.5	–	–
*Afrocarpus falcatus*	> 400	> 400	45.66 ± 5.98	–	–
*Buddleja saligna*	44.46 ± 3.46	63.43 ± 0.26	41.37 ± 5.58	0.9	0.7
*Bulbine frutescens*	> 400	> 400	304.77 ± 8.32	–	–
*Combretum apiculatum*	74.00 ± 4.48	85.94 ± 2.87	49.21 ± 0.55	0.7	0.6
*Euphorbia cooperi*	> 400	> 400	> 400	–	–
*Euphoria ingens*	> 400	> 400	> 400	–	–
*Foeniculum vulgare*	180.43 ± 4.51	357.70 ± 5.76	249.73 ± 4.72	1.4	0.7
*Galenia africana*	94.02 ± 4.21	77.99 ± 8.15	< 12.5	–	–
*Greyia radlkoferi*	96.97 ± 2.29	107.83 ± 4.84	102.59 ± 9.34	1.1	1.0
*Gunnera perpensa*	55.29 ± 9.88	130.86 ± 5.28	120.17 ± 6.29	2.2	0.9
*Myrsine africana*	> 400	> 400	> 400	–	–
*Myrothamnu flabellifolius*	244.30 ± 8.36	216.60 ± 1.61	106.33 ± 7.43	0.4	0.5
*Persicaria senegalensis*	243.60 ± 2.69	256.17 ± 3.04	118.70 ± 1.62	0.5	0.5
*Plectranthus ecklonii*	297.10 ± 34.01	340.57 ± 4.76	144.90 ± 7.15	0.4	0.4
*Tylosema esculentum* (Husk)	> 400	> 400	149.83 ± 11.83	–	–
*Tylosema esculentum* (Leaf)	> 400	> 400	> 400	–	–
Doxorubicin	0.40 ± 0.15	1.21 ± 0.08	0.63 ± 0.01	1.6	0.5
Paclitaxel l	> 400	> 400	> 400	–	–
Toxic inducer control (DMSO^a^)	9.18 ± 0.17%	9.25 ± 0.18%	2.99 ± 0.59%	0.3	0.3

^a^Dimethyl sulfoxide

^b^50% inhibitory concentration

^c^Standard deviation

^d^Selectivity index

### Anti-invasive activity

**Fig. 2:**
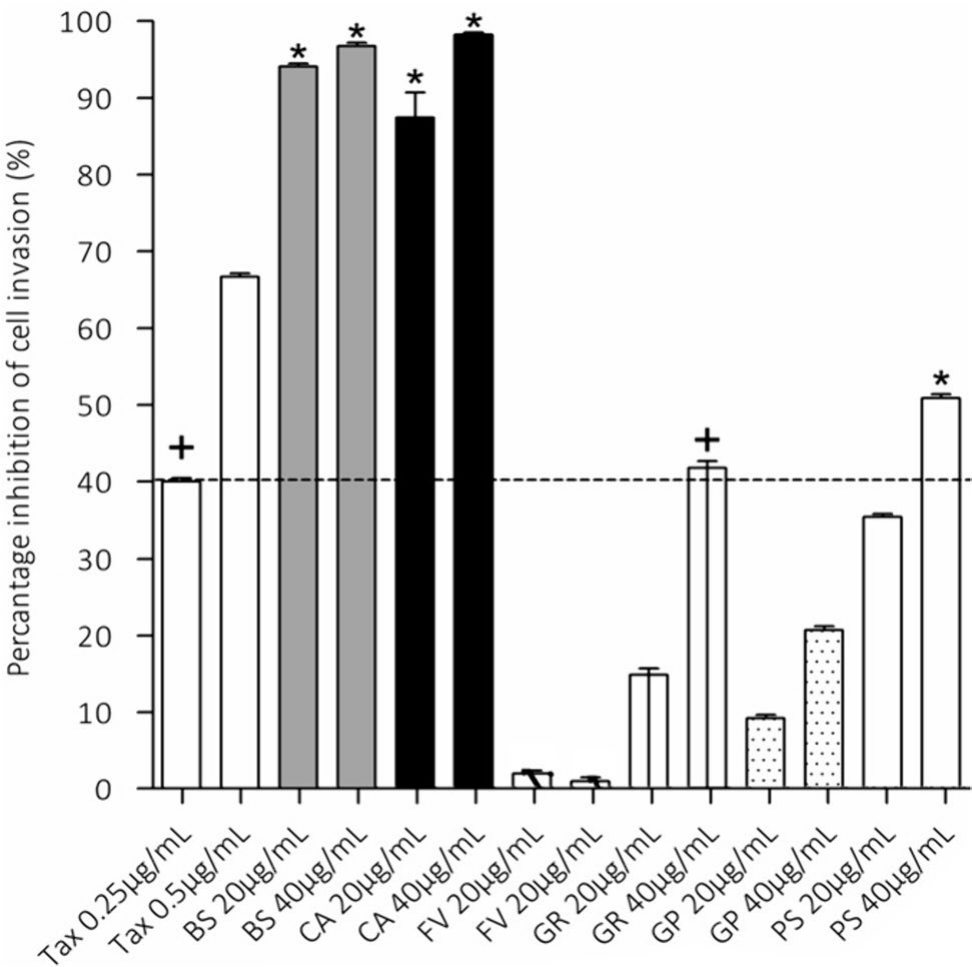
Percentage inhibition of cell invasion of *Buddleja saligna* (BS), *Compretum apiculatum* (CA), *Foeniculum vulgare* (FV), *Greyia radlkoferi* (GR), *Gunnera perpensa* (GP) and *Persicaria senegalensis* (PS) (at 20 and 40 µg/mL) and the positive control, paclitaxel (Taxol) (at 0.25 µg/mL) against invasive MDA-MB-231 breast cancer cells. Extracts were compared to Taxol (+): * indicating *p* < 0.01 showing statistical significance, and + indicating statistical similarity to Taxol (+) (one-way ANOVA, followed by Dunnett’s test)

Ethanolic extracts of *B. saligna* (BS), *C. apiculatum* (CA), *F. vulgare* (FV), *G. radlkoferi* (GR), *G. perpensa* (GP) and *P. senegalensis* (PS), were evaluated for their potential anti-invasive activity against MDA-MB-231 invasive breast cancer cells. This assay was used to quantify the percentage of cancer cells which successfully invades and migrates into the Matrigel membrane, which mimics the extra-cellular matrix between tissues, thus mimicking the invasion stage of cancer metastasis. The BS and CA extracts at both 20 and 40 µg/mL, and PS extract at 40 µg/mL only, displayed significant (p < 0.01) inhibition of cell invasion compared to the respective vehicle controls (0.1 and 0.2% DMSO). Extracts were compared to the positive control, Paclitaxel (Taxol) (+) at 0.25 µg/mL (showed 40.28 ± 6.99% inhibition of cell invasion) (Fig. 2). At both 20 and 40 µg/mL, BS (94.10 ± 0.74 and 96.73 ± 0.95% inhibition, respectively) and CA (87.42 ± 6.54 and 98.24 ± 0.63% inhibition, respectively) displayed significant inhibition of cell invasion compared to the positive control (*p* < 0.01). The FV extract displayed < 5% inhibition at both concentrations tested. The GR extract inhibited cell invasion at both 20 and 40 µg/mL (showing 14.91 ± 1.62 and 41 ± 1.78%, respectively), with inhibition at 40 µg/mL showing statistical similarity to the Taxol (+) control. The PS extract showed inhibition of cell invasion at 20 µg/mL, however, this result did not show to be significant when compared to the 0.25 µg/mL Taxol positive control. However, PS at 40 µg/mL displayed significant inhibition of cell invasion (*p* < 0.01), displaying percentage inhibition of 51.51 ± 0.83%, when compared to the 0.25 µg/mL Taxol (+) positive control. Extracts displaying significant inhibition (*p* < 0.01) at 40 µg/mL when compared to the 0.25 µg/mL Taxol (+) positive control, namely, BS (96.73 ± 0.95%), CA (98.24 ± 0.63%) and PS (51.51 ± 0.83%), were selected for further evaluation to determine their effect on the expression of CD82 in MDA-MB-231 cells.

### Effect on CD82 expression

**Fig. 3:**
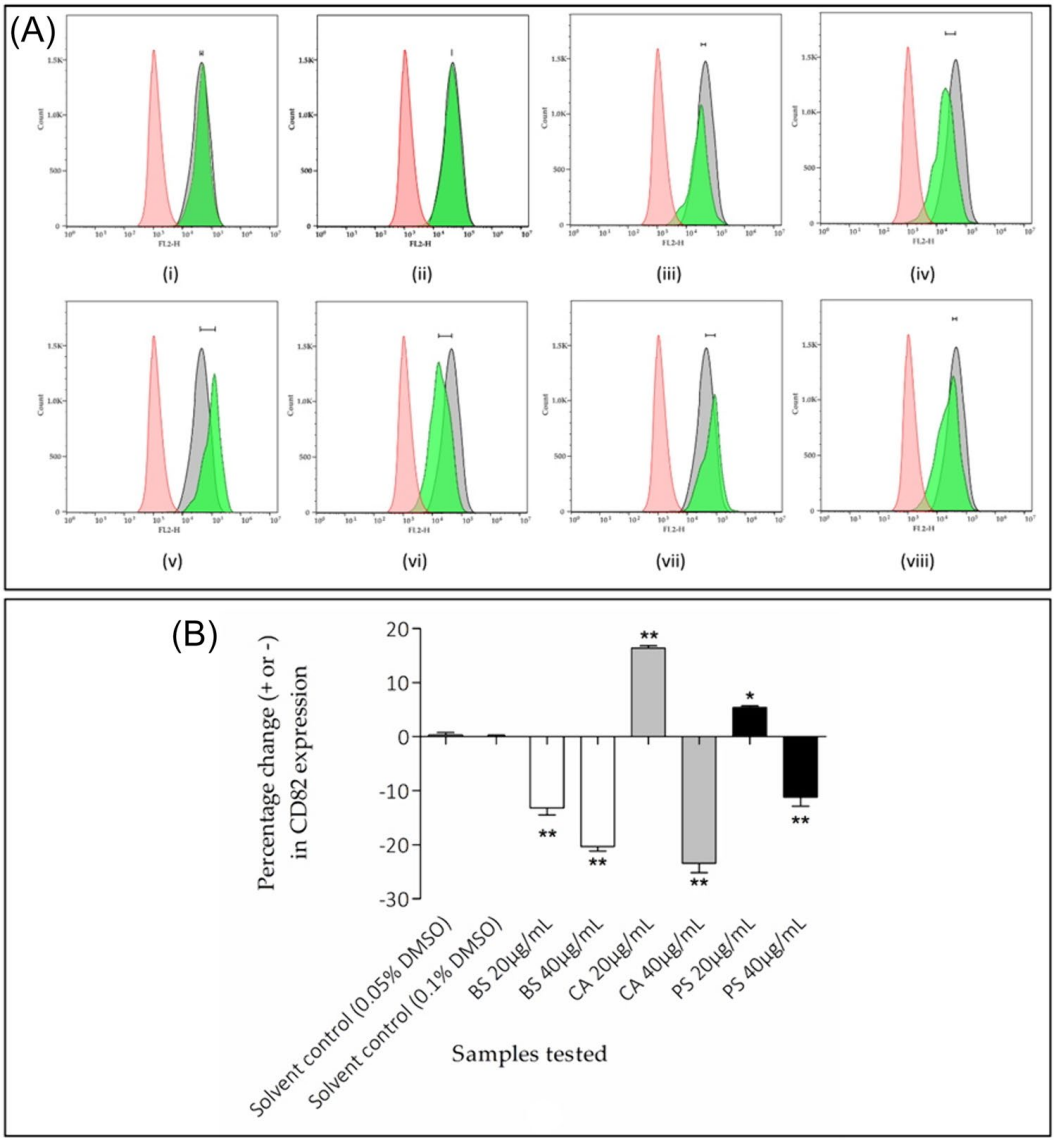
The effect of extract treatments on CD82 expression in MDA-MB-231 cells: **A** Fluorescence histograms of cells treated with (i) 0.05 and (ii) 0.1% DMSO vehicle controls, *Buddleja saligna* at (iii) 20 and (iv) 40 µg/mL, *Combretum apiculatum* at (v) 20 and (vi) 40 µg/mL, and *Persicaria senegalensis* at (vii) 20 and (viii) 40 µg/mL. Red histogram: untreated and unstained cells. Grey Histogram: untreated cells stained with CD82-PE. Green histogram: cells treated with sample and stained with CD-82 and **B** Percentage change of CD82 expression after treatment with the extracts. All samples were compared to the 0.1% DMSO vehicle control (+): * indicating *p* < 0.05 and ** indicating *p* < 0.01 showing statistical significance (one-way ANOVA, followed by Dunnett’s test)

Due to the significant anti-invasive activity of BS, CA and PS at 40 µg/mL, these extracts (at 20 and 40 μg/mL) were further evaluated for their effect on the expression of CD82 in the MDA-MB-231 cell line, after 72 h of treatment (Fig. 3). The distance between the peak fluorescence intensity exhibited by the untreated and treated cells (both stained with CD82-PE) was indicated on the fluorescence histograms (Fig. 3a). When compared to media control, the vehicle control groups (0.05% and 0.1% DMSO) showed no significant (*p* > 0.05) effect on the expression of CD82, displaying a 0.3 ± 0.8% increase and a 0.0 ± 0.5% increase in CD82 population, respectively (Fig. 3a(i, ii)). The extracts and the 0.05% DMSO vehicle control were compared to the 0.1% DMSO vehicle control (Fig. 3b). The ethanolic extract of BS significantly (*p* < 0.01) reduced CD82 expression at both concentrations of 20 and 40 µg/mL, displaying percentage decrease of 13.2 ± 2.2% and 20.3 ± 1.5% in CD82 populations, respectively (Fig. 3a(iii, iv)). Both ethanolic extracts of CA (*p* < 0.01) and PS at 20 µg/mL significantly (*p* < 0.05) increased CD82 expression (displaying 16.4 ± 0.8% and 5.4 ± 0.6% increase in CD82 populations, respectively) (Fig. 3a(v, vii)), however, both extracts at 40 µg/mL significantly (*p* < 0.01) reduced CD82 expression (displaying 23.4 ± 3.1% and 11.2 ± 2.9% reduction in CD82 populations, respectively) (Fig. 3(vi, viii)).

### Effect on in vivo angiogenesis

**Fig. 4:**
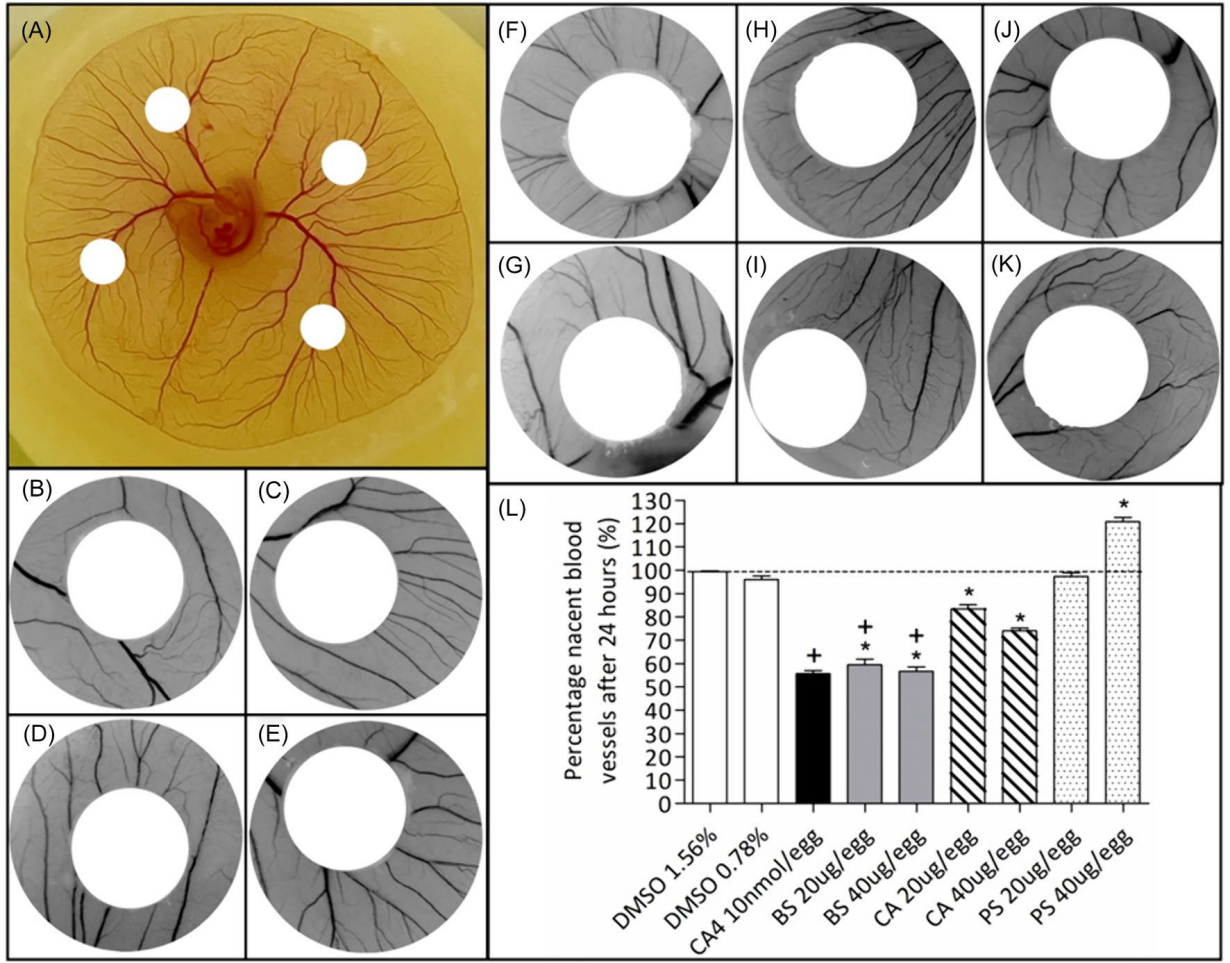
Images of **A** a chick embryo with filter paper discs placed on the membrane for sample addition. Representative images of the yolk sac membrane (YSM) treated for 24 h with **B** phosphate-buffered saline, **C** 0.78% and **D** 1.56% DMSO vehicle controls, **E** the positive control, combretastatin A4 (10 nmol/egg), *Buddleja saligna* (BS) at **F** 20 and **G** 40 µg/egg, *Combretum apiculatum* (CA) at **H** 20 and **I** 40 µg/egg, and *Persicaria senegalensis* (PS) at **J** 20 and **K** 40 µg/egg. **L** Percentage normalized nascent blood vessels (%) in YSM after treatment for 24 h. Data was expressed at mean ± SD (n = 2), where *p < 0.001, whereas (+) for BS indicates statistically similar results compared to the C4A positive control (+) (one-way ANOVA, followed by Tukey’s Multiple Comparison test)

The extracts of BS, CA and PS (at 20 and 40 μg/egg) were evaluated for their effect on in vivo angiogenesis using the YSM assay (Fig. 4). The positive control C4A at 10 nmol/egg showed 55.76 ± 3.19% newly formed blood vessels (Fig. 4E). The ethanolic extract of BS at 20 and 40 μg/egg displayed 59.52 ± 4.12 and 56.72 ± 3.13% newly formed blood vessels, respectively (Fig. 4F, G), which was statistically similar to the positive control. The ethanolic extract of CA at 20 and 40 μg/egg displayed 83.33 ± 3.17 and 74.00 ± 2.12% newly formed blood vessels (Fig. 4H, I), whereas the extract of PS at 20 and 40 μg/egg showed 97.39 ± 3.05 and 120.81 ± 3.34% newly formed blood vessels (Fig. 4J, K). One-way ANOVA compared to their respective vehicle controls (0.78 and 1.56% DMSO, displaying 96.02 ± 3.46 and 99.35 ± 0.74% nascent blood vessels, respectively) (Fig. 4B, C) showed that the CA extract significantly (*p* < 0.001) inhibited blood vessel formation at both tested concentrations, whereas the PS extract showed significant stimulation of angiogenesis (*p* < 0.001) at 40 µg/egg. Literature review shows that this is the first documented data of the pro-angiogenic potential of PS.

## Discussion

When identifying a novel agent with anti-metastatic potential, it is important to consider a candidate which displays effective antiproliferative activity, with desirable selectivity towards cancerous cells, and promotes the inhibition of cell invasive potential, via targeting the upregulation of metastatic suppressor proteins which are naturally under expressed in cancer cells.

The antiproliferative assay was used to determine the selectivity index (SI) of the extracts against cancerous and non-cancerous cells. In literature that focusses on identifying a suitable antiproliferative agent with pharmaceutical potential, the selectivity of an extract against cancerous and non-cancerous cell lines is determined based on the SI values obtained, and the following parameter is considered; the closer the selectively index of a sample is equal to one, the more favourable the drug is as this may indicate indiscriminate toxicity against both cancerous and non-cancerous cells. Ideally, a desirable antiproliferative agent for potential drug development should display selectivity towards cancerous cells over normal somatic cells (Krzywik et al. 2020). Therefore, extracts which display a selectivity index larger than or equal to one (SI ≥ 1) should be regarded as showing significant selectivity and should be considered for further evaluation. Briefly, the results from the antiproliferative data show that extracts that displayed SI ≥ 1, when comparing MDA-MB-231 to Hek293, included FV (1.4), GR (1.1) and GP (2.2), which were comparable to the SI obtained by the positive drug control, doxorubicin (1.6), whereas extracts that showed SI values ≥ 0.5 included BS (0.9), CA (0.7) and PS (0.5). Within this study, the selection criteria set for further testing of samples (SI ≥ 0.5 against both MDA-MB-231 and MCF-7 cells) was based on the lowest SI value obtained by the positive drug control, doxorubicin (SI value of 0.5 against MCF-7 cells). However, the general scientific standard for the selection of plant extracts with favourable antiproliferative activity is set to SI ≥ 1, whereas for strong anti-cancer compounds is set to SI ≥ 10 (Indrayanto et al. 2021). Therefore, future potential studies may focus on the isolation of bioactive compounds from the selected extracts and to calculate their SI values against the cell lines tested within this study, and compare them to the SI values of the extracts in order to determine if the compounds show enhanced activity against the cancerous cell lines. Comparison of the antiproliferative and cell invasion data showed a general trend where extracts with SI ≥ 1 (FV, GP and GR) showed relatively lower ability to inhibit cell invasion compared to extracts which displayed SI ≥ 0.5 (BS, CA, and PS). This may indicate that the extracts with a higher selectivity towards the breast cancer cell lines and lower inhibition of cell invasion may inhibit proliferation of cells by potentially inducing cell cycle arrest at the gap 1 (G_1_) phase, without affecting migration of cancerous cells. Literature shows that cancerous cells arrested at G_1_ phase in the cell cycle still possess invasive qualities, whilst displaying inhibition of mitosis (Kohrman and Matus 2017).

The ethanolic extract of *B. saligna* displayed the highest antiproliferative activity against all three cell lines overall, with desirable selectivity shown towards cancer cell lines. Furthermore, the BS extract significantly (*p* < 0.05) reduced cell invasion, the expression of CD82 and in vivo angiogenesis when tested at 20 and 40 µg/mL. Although BS shows multiple mechanisms of anti-metastatic inhibition, the results suggest that this may not be due to the stimulation of CD82 expression. A study conducted by Twilley et al. (2021) showed that the ethanolic leaf extract of *B. saligna* displayed antiproliferative potential, with an IC_50_ of 31.80 ± 0.35 µg/mL against UCT-MEL-1 human malignant melanoma cells. This data corresponds with an additional study by Twilley et al. (2022), which showed that the *B. saligna* extract and an isolated terpenoid mixture thereof (DT-BS-01), displayed IC_50_ values of 33.80 ± 1.02 and 5.45 ± 0.19 µg/mL, respectively, against UCT-MEL-1, and displayed respective SI values of 1.64 and 5.06 when compared to HaCat cells. This study further showed *B. saligna* and isolated compounds were able to significantly inhibit in vitro expression of vascular endothelial growth factor (VEGF) (tested at 30 µg/mL displaying 15.84 ± 4.54% inhibition) and showed significant inhibition of in vivo blood vessel growth using the YSM (showing statistically similarity to that of the C4A (+) drug control), which corresponds to the results shown within this study. The study by Twilley et al (2022) further shows the DT-BS-01 displayed comparable results to the *B. saligna* extract, and it was further determined that the mixture consisted of ursolic and oleanolic acid, which further showed to inhibit multiple pro-angiogenic factors, such as VEGF, cyclooxygenase-2 (COX-2), and interleukins interleukin (IL)-6 and IL-8. Furthermore, there are various alternative published literature which shows that oleanolic and ursolic acid display suppression of angiogenesis (Caunii et al. 2017; Chakravarti et al. 2012; Jeong 1999). Furthermore, these two compounds display multiple mechanisms of anticancer potential, including the reduction in expression (in both cancerous and non-cancerous cell lines) of various cellular factors both involved in cancer prognosis (such as Forkhead box M1 (FoxM1), cyclin D1 and cyclin-dependent kinase 4 (CDK 4), and ECM degradative enzymes (such as matrix metalloproteinases, MMP-9 and MMP-6)), or not involved in cancer prognosis (such as cytochrome p450 2e1) (Caunii et al. 2017; Chakravarti et al. 2012; Jeong 1999). An additional hypothesis may suggest that the constituents of *B. saligna* may display antiproliferative effects across all cell lines by inhibiting a general cellular pathway of standard protein expression, which is further supported by the reduction in CD82 expression observed within this study. However, further genetic and transcriptomic studies will need to be conducted on MDA-MB-231 cells treated with *B. saligna*, and its bioactive isolates, to confirm this.

The extract of *G. perpensa* displayed significant cancer selectivity against MDA-MB-231 (SI of 2.2), and significant (*p* < 0.05) ability to inhibit cell invasion at both 20 and 40 µg/mL compared to the vehicle controls, however, GP did not show significant inhibition of cell invasion when compared to the Taxol (+) positive control. Further antiproliferative research on *G. perpensa* is limited, with a study by Simelane et al. (2012), which reported that the aqueous root extract showed an IC_50_ of 222.33 μg/mL against Hek293, whereas an article by Mathibe et al. (2016) showed that the compound, Z-venusol isolated from the aqueous root extract showed an IC_50_ of 53.7 μg/mL against MCF-7 cells. This may indicate that Z-venusol may show cancer selectivity, however, there is currently no other published literature which further supports these findings. Furthermore, there is no published data on the antiproliferative activity of the ethanolic leaf extract of *G. perpensa*, nor has Z-venusol been isolated from the leaves of this species, which future studies should explore. Additionally, future research should be conducted on *G. perpensa* to identify in the species may induce cell cycle arrest at G_1_ phase.

Based on the antiproliferative data, the GR extract displayed ideal selectivity against both cancerous cell lines, and displayed significant inhibition of cell invasion at 40 µg/mL. Although, previously conducted research on the ethanolic extract of *G. radlkoferi* displayed significant inhibition of wound closure in HaCat cells using the scratch assay, displaying percentage closure of 60.15 ± 1.41% (*p* < 0.05), and 49.52 ± 1.43% (*p* < 0.01) at concentrations of 50 and 100 μg/mL, respectively (Loggenberg et al. 2022). In addition, the research also showed that the extract was nontoxic against HaCat cells at the highest concentration (cell viability > 95% at 400 µg/mL). This suggests that the leaves of *G. radlkoferi* may contain potential migrastatic compounds, which should be further isolated and investigated. A study by Lall et al. (2016) isolated and identified five compounds from the ethanolic leaf extract of *G. radlkoferi*; chalcone, galangin, genistein, pinocembrin and 2’,6’-dihydroxy-4’-methoxydihydrochalcone. There are numerous reports which display that the compounds chalcone and genistein display promising antiproliferative and anti-angiogenic activity, whereas the compounds galangin and pinocembrin display chemopreventive and anti-genotoxic mechanisms (Aderogba et al. 2012; Chien et al. 2015; Fotsis et al. 1993; Mojzis et al. 2008). Furthermore, these compounds have also shown to induce cell cycle arrest at the G_1_ phase, however, *G. radlkoferi* has yet to be evaluated for its effect on the cell cycle (Fotsis et al. 1993; Mojzis et al. 2008; Aderogba et al. 2012; Chien et al. 2015).

Although the FV extract displayed promising selectivity towards both MDA-MB-231(SI value of 1.4) and MCF-7 (SI value of 0.7) in comparison to Hek293, the extract showed no effect on cell invasion at the two concentrations tested (displaying less than < 5%). *Foeniculum vulgare* has been thoroughly investigated for its promising pharmaceutical potential and there are various literature sources which display the promising selective antiproliferative activity of *F. vulgare* against various cancerous cell lines, including breast cancer cells. Isolation studies have shown the presence of two cytotoxic compounds, such as syringin and 4-methoxycinnamyl alcohol and the presence of various compounds with antioxidant and antigenotoxic effects, such as chlorogenic acid, limonene and quercetin (Badgujar et al. 2014; He and Huang 2011; Mohamad et al. 2011). A clinical mouse model (with 4T1 Model of Breast Cancer) drug trial conducted by Mehralikhani et al. (2021) showed that aqueous decoction extract of *F. vulgare* seeds displayed tumour-reduction by increasing the ratio of the expression of E-cadherin (tumour suppressor genes) to Ki-67 and dysadherin (invasive cancer biomarkers) in the tumour tissues. Although extensively researched, the exact mechanism of action of *F. vulgare* is not fully understood, however, it may be hypothesised that this species may display cytotoxic inhibition of tumours which further decreases cancer metastatic probability, rather than decreasing the invasive potential of cancerous cells. In addition, the compounds chlorogenic and quercetin have shown to induce cell cycle arrest at G_1_, however, *F. vulgare* extract has yet to be evaluated for its effect on cell cycle arrest (Barzalona and Casanova 2008; Kim et al. 2020).

The extract of CA showed strong antiproliferative ability against all three cell lines, displayed significant (*p* < 0.01) inhibition of cell invasion against MDA-MB-231 at both at both 20 and 40 µg/mL, displayed a significant (*p* < 0.05) increase in CD82 populations at 20 µg/mL and significantly (*p* < 0.01) decreased CD82 expression at 40 µg/mL. It may be hypothesised that CA displays promising inhibition of cell invasive potential in MDA-MB-231, with a potential target mechanism being the upregulation of expression of CD82 protein, when tested at 20 µg/mL. However, conflicting results from the YSM assay showed CA tested at both 20 and 40 µg/egg displayed significant inhibition of nascent blood vessels formation. These results and literature may suggest that the inhibition of angiogenesis by CA may not be influenced by the downregulation of CD82, and rather by an alternative mechanism, such as the down regulation of VEGF, as angiogenesis was inhibited at both concentrations. There is limited research investigating the antiproliferative and anticancer potential of *C. apiculatum*, where only one research article by Maphutha et al. (2021) displayed the inhibitory activity of the ethanolic leaf extract of *C. apiculatum* against A431 and UCT-MEL-1 skin cancer cells, exhibiting IC_50_ values of 56.40 ± 6.11 and 90.53 ± 4.94 µg/mL, respectively. A study by Fyhrquist et al. (2006) showed that the methanolic leaf extract of *C. apiculatum* exhibited IC_50_ values of 65.0 ± 17.0 and 40.1 ± 6.8 µg/mL against T 24 bladder cancer and MCF-7 cells, whereas the methanolic root extract displayed IC_50_ values of 30.9 ± 1.9, 58.1 ± 0.56, and 44.0 ± 5.7 µg/mL. Furthermore, various other *Combretum spp.* have displayed varying activity against human cancer cell lines (Fyhrquist et al. 2006), and numerous antiproliferative compounds, such as cardamonin, pinocembrin, quercetin, and kaempferol, have been isolated from *C. apiculatum* (Aderogba et al. 2012). Although the potential effect of *C. apiculatum* on angiogenesis has not been previously evaluated, isolated compounds from the species have displayed inhibition of angiogenesis by targeting VEGF receptor-2 (Aderogba et al. 2012; Kim et al. 2020; Osonga et al. 2019; Sharma et al. 2018; Xueni Wang et al. 2019a, b). However, further in vitro analysis has yet to be conducted to confirm this mechanism of action for *C. apiculatum.* Furthermore, it is also important to note that the CA4 positive control used in this study is a potent anticancer compound which was previously isolated from the bark of *Combretum caffrum* (Eckl. & Zeyh.) Kuntze, a species related to *C. apiculatum* (Pettit et al. 1989). Literature shows that combretastatin compounds display potent anticancer activity and have been found in various *Combretum spp* (Fyhrquist et al. 2006). However, isolation and identification of these compounds in *C. apiculatum* has yet to be conducted. Furthermore, the extract of CA, and isolation of bioactive compounds, should be considered for further evaluation for anti-metastatic potential by targeting pro-angiogenic factors, such as VEGF, COX-2 and interleukins IL-6 and IL8.

The extract of PS displayed varying activity against the three cell lines, showing significant (*p* < 0.05) inhibition of cell invasion against MDA-MB-231 at 40 µg/mL, and stimulation of CD82 at 20 µg/mL, whereas PS at 40 µg/mL showed a significant (*p* < 0.05) decrease in CD82 expression. Furthermore, results from the YSM assay showed that PS significantly (*p* < 0.001) stimulated angiogenesis only when tested at 40 µg/mL. Therefore, the results suggest that PS showed the least antiproliferative activity, but similar to CA, stimulated CD82 and inhibited in vivo angiogenesis at 20 µg/mL. However, at 40 µg/mL, PS showed an inhibition of CD82, with a resulting increase in angiogenesis, indicating that targeting CD82 may be the major mode of action for PS, as a change in activity of CD82 showed a direct effect on angiogenesis. Literature shows that there is limited, yet promising, antiproliferative activity of *P. senegalensis*, with only one study by Mohamed et al. (2018) that showed the methanolic leaf extract and methanolic seed extract of *P. senegalensis* displayed activity against PC3 (IC_50_ values of 2.3 ± 0.03 and 3.5 ± 0.06 µg/mL, respectively) and Caco-2 colon cancer cells (IC_50_ values of 2.0 ± 0.03 and 1.5 ± 0.03 μg/mL, respectively). However, further isolation and in vitro analysis should be conducted on *P. senegalensis* to identify the potential mechanism of antiproliferative activity, such as the effect of PS on growth factors (such as platelet derived growth factor (PDGF-AA)). Furthermore, the YSM data suggests that PS may possess proangiogenic mechanisms of action, separate to that associated with inhibition of cell proliferation, such as by stimulation of COX or VEGF. Therefore, future studies conducted on *P. senegalensis* should focus on identifying the bioactive compounds respectively responsible for the antiproliferative and proangiogenic effects displayed by the species. Alternatively, the antiproliferative and proangiogenic effect of PS suggests that the extract may display stimulation of an alternative molecular marker involved in the stabilization of angiogenesis, such as elastin. However, further testing will need to be conducted to confirm this.

Various clinical trial studies, involving cases of breast cancer diagnosed at varying stages, has shown that upregulated CD82 protein expression correlates with less malignant cases of diagnosed breast cancer, whereas down regulated CD82 protein expression shows correlation with highly malignant cases of diagnosed breast cancer (Al-Khater et al. 2021; Jee et al. 2006; Singh et al. 2016; Wang et al. 2019a, b). Therefore, samples shown to upregulate the expression of CD82, such as PS, but display conflicting anti-invasive and anti-angiogenic results, should be subjected to bio-assay guided fractionation to identify bioactive compounds which may display inhibition of cell invasion and angiogenesis, by the upregulation of CD82. Furthermore, literature shows that extracts of *B. saligna*, *F. vulgare* and *G. perpensa* have shown to be non-mutagenic by Microbial Ames Mutagenicity assay, whereas the potential mutagenicity of *C. apiculatum, G. radlkoferi* and *P. senegalensis* have yet to be evaluated (Badgujar et al. 2014; Shirinda et al. 2019; Twilley et al. 2021). In addition to this, none of the aforementioned species have been subjected to in vivo toxicological studies. Therefore, these extracts and isolated compounds should be evaluated in future to determine their potential safety as candidates for future clinical drug trials. In addition, the study focussed on evaluating extracts solely extracted by use of absolute ethanol, as opposed to alternative extraction solvent mixtures which may potentially result in higher extraction yields of polyphenols with potential bioactivity. Therefore, another potential avenue for future studies may focus on the comparison of bioactivity of the plant extracts produced by extraction with alternative organic solvent mixtures, such as methanol, or aqueous-ethanol mixtures. Furthermore, future research should focus on the isolation and identification of the bioactive compounds which may be responsible for the respective bioactivities shown within this study. In addition to this, further comparison studies should be conducted to discuss the potential differences in bioactivity and selectivity between the crude extracts, and their respective isolated compounds.

## Conclusion

When identifying a promising potential anti-metastatic agent against breast cancer, it is important to identify candidates which display adequate antiproliferative selectivity (SI closer to, or larger than 1.0), and significant ability to inhibit cell invasion and angiogenesis, by targeting the upregulation of expression of CD82.The extracts displayed varying antiproliferative activity against MDA-MB-231, MCF-7 and Hek293 cells, with extracts of BS (0.9), CA (0.7), FV (1.4), GR (1.1), GP (2.2), and PS (0.5) displaying promising selectivity (SI ≥ 0.5) towards MDA-MB-231 when compared to Hek293. It was hypothesised that extracts displaying high selectivity but low ability to inhibit cell invasion may inhibit mitosis independently from affecting cell migration, potentially by inducing cell cycle arrest at G_1_ phase. The FV extract displayed promising selectivity but did not show inhibition of cell invasion (when tested at 20 and 40 µg/mL), whereas the GP and GR extract displayed promising selectivity against MDA-MB-231 cells, and inhibited cell invasion (at both at 20 and 40 µg/mL), albeit not showing significance compared to taxol (+). Furthermore, the extracts of GP and GR, and isolated compounds thereof, should be considered for further in vitro evaluation for antiangiogenic potential, such as the in vitro effect on growth factor expression (namely, VEGF). The extract of BS and CA displayed promising antiproliferative selectivity, and when tested at both 20 and 40 µg/mL, showed significant inhibition of cell invasion and in vivo angiogenesis (with BS showing statistical similarity to the CA4 positive control). However, BS showed a significant decrease in expression of CD82 at both 20 and 40 µg/mL, whereas CA showed a significant increase in CD82 expression at 20 µg/mL, and significantly decreased CD82 expression at 40 µg/mL. These results suggest that BS may display multi-functional inhibition of various molecular factors, in addition to CD82, which may be responsible for the facilitation of angiogenesis. However, further studies will need to be conducted to support this, such as the evaluation of the effect of BS on other angiogenic factors. With regards to the CA extract, isolation studies have yet to be conducted to identify potential anticancer compounds, and further literature review shows that CA has not been evaluated for the presence of combretastatin compounds isolated from other species of the *Combretum* genus (Maphutha et al. 2021). Furthermore, additional mechanistic studies should be conducted to determine the potential alternative mode of action by which CA inhibits angiogenesis. The PS extract displayed adequate selectivity, and significantly inhibited cell invasion at 40 µg/mL. Furthermore, PS significantly decreased CD82 expression at 20 µg/mL, however, when tested at 40 µg/mL showed a significant reduction in CD82 expression and significantly stimulated in vivo angiogenesis. Therefore, isolation of bioactive compounds from the extracts of CA, BS and PS should be considered to identify compounds which may be separately responsible for the proangiogenic activity, antiproliferative activity, and stimulation of CD82 expression. Furthermore, extracts of CA and PS showed promise to be suitable plant candidates for the upregulation of CD82 expression at the lower tested concentration, with differing effects on angiogenesis at the two concentrations tested. Lastly, CA displayed promising in vitro potential against cellular factors related to stages of metastasis, with the ability to stimulate CD82 in breast cancer cells and inhibit in vivo angiogenesis. Therefore, further isolation of bioactive compounds and pre-clinical testing should be considered for CA and PS to identify the compounds responsible for the stimulation of CD82, and correlating anti-invasive activity, as a novel therapeutic mechanism against breast cancer. Furthermore, gaps in literature show that the mutagenicity of CA, GR and PS, and potential in vivo toxicology of BS, CA, FV, GP, GR and PS have yet to be evaluated, and should therefore be investigated in future study to validate the safety of these plant candidates in therapeutic use.

## Data Availability

The datasets generated during and/or analysed during the current study are available from the corresponding author on reasonable request.

## Abbreviations

BS: *Buddleja saligna* Willd.
CA: *Combretum apiculatum* Sond.
CA4: Combretastatin A-4
CANSA: Cancer Association of South Africa
CDK 4: Cyclin-dependent kinase 4
CD82: Cluster of differentiation 82 metastatic suppressor protein
COX-2: Cyclooxygenase 2
CSB: Cell Staining Buffer
DMEM: Dulbecco’s Modified Eagle Medium
DMSO: Dimethyl sulfoxide
ECM: Extracellular matrix
EtOH: Ethanol
FBS: Fetal bovine serum
FoxM1: Forkhead box M1
FV: *Foeniculum vulgare* Mill.
GP: *Gunnera perpensa* L.
GR: *Greyia radlkoferi* Szyszył.
G_1_: Gap 1 phase
Hek293: Human kidney cell line
IC_50_: Half maximal inhibitory concentration
IL: Interleukin
MCF-7: Non-invasive breast cancer cell line
MDA-MB-231: Invasive breast cancer cell line
MMP: Matrix-metalloproteinase
PS: *Persicaria senegalensis* (Meisn.) Soják
SI: Selectivity index
VEGF: Vascular endothelial growth factor
YSM: Egg yolk sac membrane assay
